# Efficacy of the Applied Natural Enemies on the Survival of Colorado Potato Beetle Adults

**DOI:** 10.3390/insects12111030

**Published:** 2021-11-16

**Authors:** Vladimír Půža, Jiří Nermuť, Jana Konopická, Oxana Skoková Habuštová

**Affiliations:** Biology Centre, Czech Academy of Sciences, Institute of Entomology, Branišovská 31, 370 05 České Budějovice, Czech Republic; Jirka.Nermut@seznam.cz (J.N.); jkonopicka@seznam.cz (J.K.); habustova@entu.cas.cz (O.S.H.)

**Keywords:** Colorado potato beetle, entomopathogenic nematodes, entomopathogenic fungi, *Steinernema*, *Beauveria*, field application

## Abstract

**Simple Summary:**

Colorado potato beetle (CPB) *Leptinotarsa decemlineata* is the potato plant’s most destructive pest. Recently, resistance to the traditional insecticides has appeared, thus new environmentally friendly control agents are highly needed. In our study, we searched for the most effective entomopathogenic agents that could be used to decrease the emergence of CPB adults from the soil. We selected two entomopathogenic nematodes (*Steinernema carpocapsae* and *S. feltiae*) and one strain of fungus (*Beauveria bassiana*). The suspension application was done on the leaves, plus by watering the pods and the field plots. All the treatments had an obvious effect, but in the field, only the fungal treatment showed a promising result. Further research is needed to develop the most effective application for field usage.

**Abstract:**

Colorado potato beetle *Leptinotarsa decemlineata* is among the most destructive pests of potatoes quickly developing resistance to traditional insecticides. In the present study, we tested the effect of various species and strains of entomopathogenic nematodes on CPB adults, and subsequently, the most effective nematodes were applied alone and in combination with entomopathogenic fungus *B. bassiana* in pots with potato plants and in the field and their effect on the number of emerging adults was evaluated. In the experimental infections, both the nematode invasion and pathogenicity were variable, and, in several strains, the mortality reached 100%. In pot experiments, soil application of nematodes *S. carpocapsae* 1343 and *S. feltiae* Jakub and fungus significantly decreased numbers of emerging CPB adults, while, after the application on leaves, only fungal treatment was effective. The field application of fungus *B. bassiana* significantly decreased the number of emerging CPB adults in comparison to control sites by ca. 30% while the effect of nematodes and the nematodes–fungus combination was not significant. In conclusion, we demonstrate the necessity of thorough bioassays to select the most effective nematode strains. Entomopathogenic nematodes have the potential to effectively decrease the emergence of CPB adults, but further research is needed to improve the effectiveness in the field.

## 1. Introduction

Colorado potato beetle (CPB), *Leptinotarsa decemlineata* Say (Coleoptera: Chrysomelidae), is among the most destructive pests of potatoes (*Solanum tuberosum* L.). The adults and larvae feed on potato plants, significantly reducing the yield [1]. Since CPB is known to develop resistance to traditional insecticides [2,3,4], new environmentally safe control strategies are highly needed.

Entomopathogenic nematodes (EPNs) (Steinernematidae and Heterorhabditidae: Nematoda) are ubiquitous lethal insect parasites with global distribution and a wide host range [5]. Due to their ability to infect various insects [6], the possibility of mass production by industrial techniques [7], and their safety to non-target organisms and the environment [8,9], EPNs represent an attractive agent for the biological control of many insect pests [10].

Entomopathogenic fungi (EPFs) represent other promising biocontrol agents. Their advantages are that they do not need to be ingested, as they are able to penetrate the host cuticle, and that they can be relatively easily produced [11]. The fungi are produced either in solid-state fermentation, where they produce aerial conidia, or in a submerged liquid-state fermentation, where they produce blastopores [12], and a number of mycoinsecticide, have been developed in the world [13].

In the last three decades, a considerable number of studies addressed the potential use of entomopathogenic nematodes for control of CPB, but with variable results. Under laboratory conditions, Cantelo and Nickle [14] tested three EPN species at different dosage levels and achieved 100% mortality of CPB prepupae at a dose of 164.6 nematodes/cm^2^. Similarly, Trdan et al. [15] have shown that four EPN species caused the prompt death of all CPB stages at doses of 200, 1000, and 2000 infective juveniles per individual in Petri dish experiments. So far only several field studies were performed. Nematode *Heterorhabditis marelatus* applied twice during the season caused a 50% reduction in adult CPB populations and produced six times as many dead prepupae in nematode-treated soil samples as in the untreated samples [16]. Recently, Čačija et al. [17] have demonstrated that *S. feltiae* and *S. carpocapsae* effectively decreased the number of overwintering CPB adults.

The effect of control agents can be increased by their combination [18,19]. The combined application of the nematode *S. feltiae* with entomopathogenic fungus *Isaria fumosorosea* significantly increased the CPB mortality in comparison to single-agent treatment [20]. Özdemir et al. [21] observed a synergistic effect between several chemical insecticides and *S. feltiae* against CPB. On the other hand, the negative effect of the combination has been recorded in the interactions between the EPN *S. glaseri* and fungus *Metarhizium anisopliae* [22] and between *Steinernema ichnusae* and *B. bassiana* fungus [23]. Therefore, the compatibility of control agents should be thoroughly tested before large-scale applications.

Colorado potato beetle adults are generally less susceptible to nematodes [16]. However, the reduction of pupating adults is desirable as it could decrease the damage caused by second-generation CPB in field conditions, as well as adult emergence in the next spring. Therefore in the present study, we focused on adult CPB and we tested a large number of EPN species and strains for pathogenicity to CPB adults in Petri dish assays in order to select the most effective nematodes. The selected nematodes were tested alone and in combination with entomopathogenic fungus in pot and field experiments. The combined application allows the testing of the hypothesis that the combination of the nematodes with EPF could increase the effectivity against CPB.

## 2. Materials and Methods

### 2.1. Culture of Colorado Potato Beetle

The adults and larvae of Colorado potato beetle (CPB) were collected from the potato plants on an organic farm near the vicinity of Malonty village, Czech Republic (48.7083920 N; 14.5778508 E) in several consecutive series in July and August 2019–2021. Each developmental stage (larvae and adults) was collected separately inside a plastic box of size 34 × 22.5 × 15.7 cm with a perforated lid.

The collected culture of CPB was placed inside the fine mesh cage of size 100 × 50 × 50 cm. Potato plants of the variety Magda, grown in a pot with a diameter of 21 cm and a volume of 4 L, were provided for feeding. Culture was left in controlled greenhouse conditions (25 °C, 75% relative humidity and a long day photoperiod 16:8). Culture was checked daily and supplemented with fresh potato plants. Potato plants of the variety Magda were obtained in the form of tubers and tissue cultures (tiny plants) from the Potato Research Institute, Havlíčkův Brod. Plants were grown in a pot with a diameter of 21 cm and a volume of 4 L, watered daily, and left in the same conditions as a CPB culture. The Magda variety is a dynamically growing plant with a big leaf and, therefore, is highly suitable for feeding.

### 2.2. Rearing of Nematodes

The nematodes used in this study either originated from the collection of the Laboratory of Entomopathogenic Nematodes, Biology Centre CAS, which contains both Czech and exotic EPN strains, or were obtained from the field sampling performed during this research (Table 1). In total, 32 EPN strains were used. Soil samples were collected in potato fields and field edges in 15 localities in Czech Republic in the years 2019 and 2020. At each sampling site, five samples of approximately 1 kg of soil were taken with a hand shovel to a depth of ca. 20 cm. Each sample consisted of 10 subsamples from a ca. 100 m^2^ area. The modified Galleria baiting technique of Bedding and Akhurst [24] was used for EPN isolation. Four last-instar *G. mellonella* larvae were added to each sample. Samples were incubated for 5 days at 20 °C and at 55% relative humidity. Dead larvae were individually incubated in the White Traps (White, 1927). The infective juveniles (IJs) recovered from the traps were used both for molecular identification and the subsequent experiments. The nematode strains in the collection are maintained according to Kaya and Stock [25]. Before the experiments, all nematodes were propagated using *G. mellonella*, and 2–3-week-old IJs were used for the infections.

Mass Rearing of Nematodes for Field Application

Nematodes for the field experiment were monoxenically mass produced in liquid medium by modified method according Stock and Goodrich-Blair [26] as follows. Gravid females of *Steinernema feltiae*, strain Jakub, were obtained by the dissection of *Galleria mellonella* larvae infected 4 days before dissection. Females (ca. 50 individuals) were placed in the 1.5 mL Eppendorf tubes with sterile tap water. Later, the water was removed, and females were gently crushed by a plastic microhomogenizer directly in the tube. Water was added (1 mL) to the homogenized females, and the tubes with females were centrifuged for 2 min. at 2000 rpm. After this step, eggs were concentrated in the form of a small but clearly visible pellet on the bottom of the tubes. The supernatant was removed by pipet, and cleaning with water was repeated once again. After that, 1 mL of sterilization solution (10 mL tap water, 1.5 mL 4 M NaOH, and 0.5 mL 12% NaOCl) was added. Eggs were incubated in this solution for 4 min., and after that, the tubes were centrifuged for 2 min. at 4000 rpm to concentrate the eggs into a pellet again. The sterilization solution was removed and replaced by 1 mL of YS medium (5 g yeast extract, 5 g NaCl, 0.5 g NH_4_H_2_PO_4_, 0.5 g K_2_HPO_4_, 0.2 g MgSO_4_·7H_2_O, 1 L distilled water), then centrifuged again for 2 min. at 4000 rpm. This wash with YS medium was repeated twice. After that, sterile YS in a volume of 1 mL was added and the solution of medium with eggs was transferred to sterile multiwell plates (24 wells per plate), with 300 µL of solution per one well. Plates were then sealed with parafilm and stored at 16 °C for 72 h in a climatic box. If the solution was without any turbidity after that time, eggs or the hatched first stage larvae were considered sterile and axenic.

Symbiotic bacteria for the mass production of nematodes were obtained from *Galleria mellonella* larvae 24 h after infection with an appropriate nematode strain. Firstly, the infected larvae were washed in 70% ethanol and dried, and then one leg was cut by sterile scissors. A drop of hemolymph was placed on NBTA agar (37 g standard nutrient agar I, 25 mb bromthymolblue, 4 mL 1% 2,3,5,-triphenyl-tetracoliumcholoride) plate and dispersed on the surface of the plate; 90 mm Petri dishes with bacteria were sealed with parafilm and stored at room temperature, ca. 22 °C. One day later, a single colony of the growing bacteria was transferred to the sterile YS medium (20 mL of medium in a 50 mL Erlenmeyer flask) and cultivated with a shaker at 180 rpm for 2 days. One ml of two-day-old culture was then used as inoculum for sterile nematode culture medium (50 mL of medium in a 250 mL Erlenmeyer flask), composed according to Dunn et al. [27]: 15 g yeast extract, 20 g soy powder, 4 g NaCl, 0.35 g KCl, 0.15 g CaCl_2_, 0.1 g MgSO_4_, 36 mL olive oil, and 1 L distilled water.).

Nematode culture medium inoculated with bacteria was shaken at 180 rpm at 18 °C for 48 h, and after that time, axenic nematode larvae were added to the flask. By this method, the established monoxenic culture was cultivated for 14 days at 18 °C and 160 rpm. Within this time, the nematodes finished their life cycle and produced new IJs on a mass scale. These IJs were then used as inoculum for the subsequent mass production that was performed as described before. The nematode culture medium pre-inoculated with bacteria was settled with 1 mL of IJs obtained from the first cultivation. After 14 days, we collected nematodes for field applications and other experimental purposes. Nematodes were collected with the simple sedimentation method and by washing in tap water. All of the work regarding the cultivation of nematodes was done in strictly sterile conditions in a UV-sterilized flow box.

### 2.3. Rearing of Entomopathogenic Fungus

*Beauveria bassiana* strain BBA 08 was used in this study. The strain was isolated from the adult of the Colorado potato beetle (CPB), *Leptinotarsa decemlineata,* from the Bělčice site in the Czech Republic (49.50702 N; 13.89545 E). The strain was identified on the basis of macroscopic, microscopic, and genetic characteristics and has been deposited at the Biology Centre CAS, České Budějovice. The GenBank accession number of the LSU sequence is MN749315.

Blastospores of the fungus BBA 08 were used in the experiments. The fungus was cultured in Potato Dextrose Broth liquid medium (Sigma-Aldrich, Darmstadt, Germany) at 25 ± 1 °C. For cultivation, 95 mL of liquid media was put into a 250 mL Erlenmeyer flask and inoculated with 5 mL of conidial fungus suspension. Then the flask was placed on an orbital shaker with a speed of 200 rpm and temperature 25 ± 1 °C for four days. After four days, the suspension was filtered through sterile gauze to separate the mycelium. In uniform suspension, the spores were counted with a Neubauer improved counting chamber (Sigma-Aldrich, Darmstadt, Germany), and subsequently, the suspension was adjusted to the required concentration.

The viability of spores was verified using a standard germination test [28]. Ten drops from suspension were applied using a 1 μL inoculation loop on the surface of 2% water agar, which was poured in a thin layer onto the surface of a sterile slide. After the drops had dried, the slides were moved into a wet chamber and incubated at a temperature of 25 ± 1 °C for 24 h. The percentage of germinating spores was determined using an Olympus CH20 light microscope (Olympus Optical Co., Ltd., Tokyo, Japan); bright field, 400× magnification. The spore germination in all tests was 100%.

### 2.4. Petri Dish Infections

In the experimental infections, the CPB adults were individually infected in Petri dishes (9 cm diameter) lined with moist filter paper and a potato leaf for feeding. Various collection and freshly isolated EPN strains (Table 1) were applied at three doses of 250, 500, and 1000 IJs per one dish in a total water volume of 450 µL, each dose being applied in ten replicates. Control dishes received 450 µL of water only, and there were ten control dishes for each nematode strain. The mortality of CPB was observed for 6 days and then both dead and live beetles were dissected in order to check for the presence of nematodes.

### 2.5. Pot Experiments

Pot experiments were designed to confirm the effectivity of the selected nematodes and the entomopathogenic fungus *B. bassiana* strain BBA 08 in outdoor conditions prior to the field application. Based on the Petri dish experiment, highly pathogenic strains *S. feltiae* strain Jakub and *S. carpocapsae* strain 1343 were selected. Some other strains displayed comparable, or even slightly higher pathogenicity, but we selected these Czech strains as they could be better adapted to local outdoor conditions.

The effects of both nematodes and fungus *B. bassiana* strain BBA 08 on pupating Colorado potato beetles in the soil and on CPB adults on plant leaves were tested in pots with potato plants (see above) in outdoor conditions, in České Budějovice, Czech Republic (48.9764494 N; 14.4473356 E).

Each combination was tested in 8 plastic pots (diameter 21 cm, 15 cm of organic soil) with one potato plant per pot (25 cm high) that was populated with 5 IV. instar larvae or 5 adults of *L. decemlineata* prior to the application. Another four pots with either the IV. instar CPB larvae or adults were prepared as the control without bioagent application.

To assess the effect on pupating CPB, both nematodes and fungus were applied to the soil by automatic pipette in 5 mL of water at a dose of 21,000 IJs for the nematodes and 1 × 10^9^ for the fungus with nematode dosage corresponding to the standard recommended doses (5 × 10^5^ IJs per square meter) and fungal dose corresponding to the half of the highest recommended dose for a commercial product based on *B. bassiana* BOTANIGARD 22 WP (5.37 × 10^10^/m^2^). In the experiment with adults on leaves, the nematodes and fungus in the same dosage were sprayed on plant leaves. Each pot was closed into an entomological isolator to prevent adult beetles and larvae from escaping and also to protect them from natural enemies from the surrounding area.

The effect of leaf application was observed every day and after one week the final evaluation was done when all dead and live adults were counted. To evaluate the effect of soil application, the larvae were allowed to leave the leaves and move to the soil to pupate. Then the pots were observed daily and the numbers of emerging adult beetles were recorded.

### 2.6. Field Application

The field trial area was located near the village of Žabčice in the South Moravian Region (49.0219636 N; 16.6155681 E), in the corn production area, altitude 178 m, average air temperature (1991–2020) 10.3 °C, average total precipitation (1991–2020) 491.1 mm. The surface is dominated by fluvial gley, and the soil type is a clayey loam. The pre-crop was spring barley. Our field was prepared with standard agrotechnical methods.

**Figure 1:**
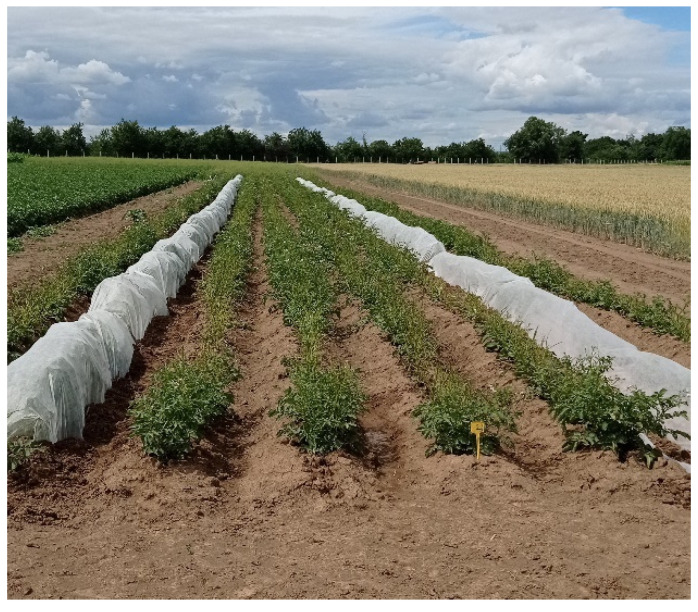
Experimental field with experimental rows covered with non-woven fabric.

The total experimental area was 302.4 m^2^, which was divided into 12 experimental plots by using GPS navigation (Figure 1). One experimental plot (25.2 m^2^) contained 4 rows of potatoes, and the length of the row was 8.4 m; the number of tubers in a row was 28; the distance between tubers in a row was 30 cm, and the pitch was 0.75 cm. The planted variety of potatoes was Rosara. Entomopathogenic nematode *S. feltiae*, strain Jakub, was selected as the more effective nematode from the pot experiments. The application of nematodes, fungus *B. bassiana* strain BBA 08, their combination, and control was done shortly before the larvae of IV instar were ready to climb for pupation into the soil (larvae from the first generation of CPB adults). Each agent application was repeated in 3 repetitions.

The bioagents were applied at doses of 12.5 million IJs of EPN/plot/40 L of water, 1.72 × 10^11^ spores of EPF/plot/40 L of water, and 12.5 million IJs of EPN + 1.72 × 10^11^ EPF/plot/40 L. The nematode dose was derived from the recommended dose (5 × 10^6^ IJs per 10 m^2^) and the dose of EPF corresponded to ca. 12% of the highest recommended dose for the commercial product *B. bassiana* BOTANIGARD 22 WP (5.37 × 10^10^/m^2^).

The control plots were watered by 40 L of water/plot. After application, two rows along the entire field trial were randomly selected, and treated plots were separately covered with non-woven fabric to prevent the leakage and mixing of CPBs (Figure 1). After 14 days, all second-generation CPB adults were counted. The occurrence was compared between treated plots with different bioagents and their combination to the control experiments.

### 2.7. Statistical Analyses

All statistical analyses were performed in Statistica version program 10, StatSoft Inc., Tulsa, OK, USA. Main effect ANOVA was used to analyze the data for invasion (numbers of invaded nematodes) and mortality in Petri dish experiments and for numbers of emerging beetles in the field experiment. One-way ANOVA was used to compare the numbers of emerging adults after soil application in the pot experiment and for the mortality data after leaf application and to compare invasion and mortality between endemic and non-endemic nematode strains. Tukey test was used to detect differences among individual treatments in the pot experiments. Prior to the analyses, count data (nematode invasion, numbers of emerging CPB adults) were square root transformed, and arcsin transformation was used for mortality data. In the text, the data are presented in the form of mean ± SEM.

## 3. Results

### 3.1. Field Sampling

The sampling in the potato fields resulted in the isolation of 21 steinernematid strains (Table 1), out of which 13 strains belonged to *S. feltiae*, 6 strains were identified as *S. affine,* and 2 strains belonged to *S. kraussei*. The nematodes were present both on the field margins and within the fields.

### 3.2. Petri Dish Infections

During the dissections, we could observe that part of the nematodes were dead and encapsulated by CPB hemocytes. Nematodes were present also in the living insects, where dead nematodes prevailed. The proportion of dead nematodes also differed among nematode strains and the development to first-generation adults was observed only in some strains (data not shown). Mortality in control dishes was negligible.

**Figure 2:**
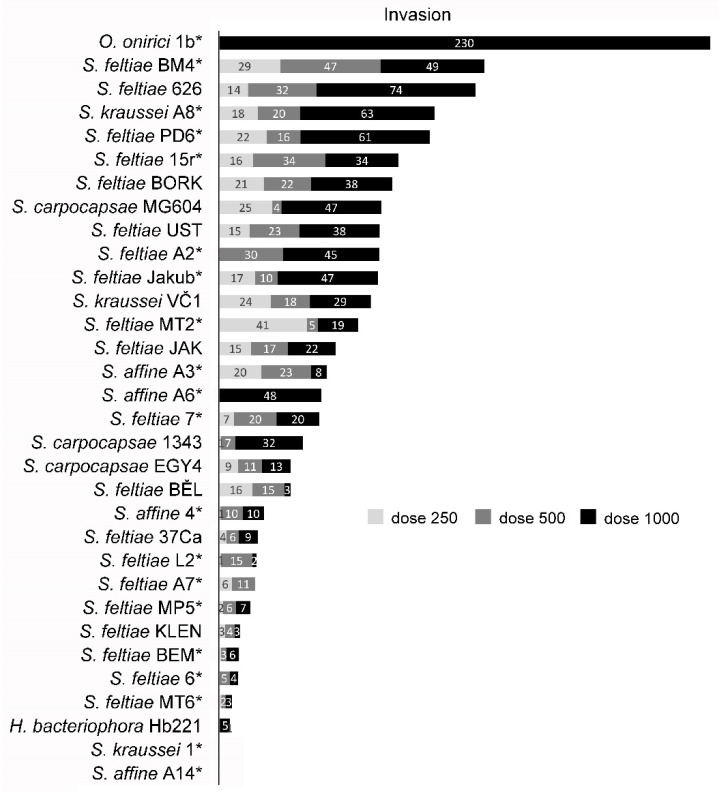
Mean numbers of nematodes of various EPN species and strains that invaded in adult Colorado potato beetles. The nematodes were applied at three doses of 250, 500, and 1000 IJs per host. Endemic strains isolated within this study are marked (*).

**Figure 3:**
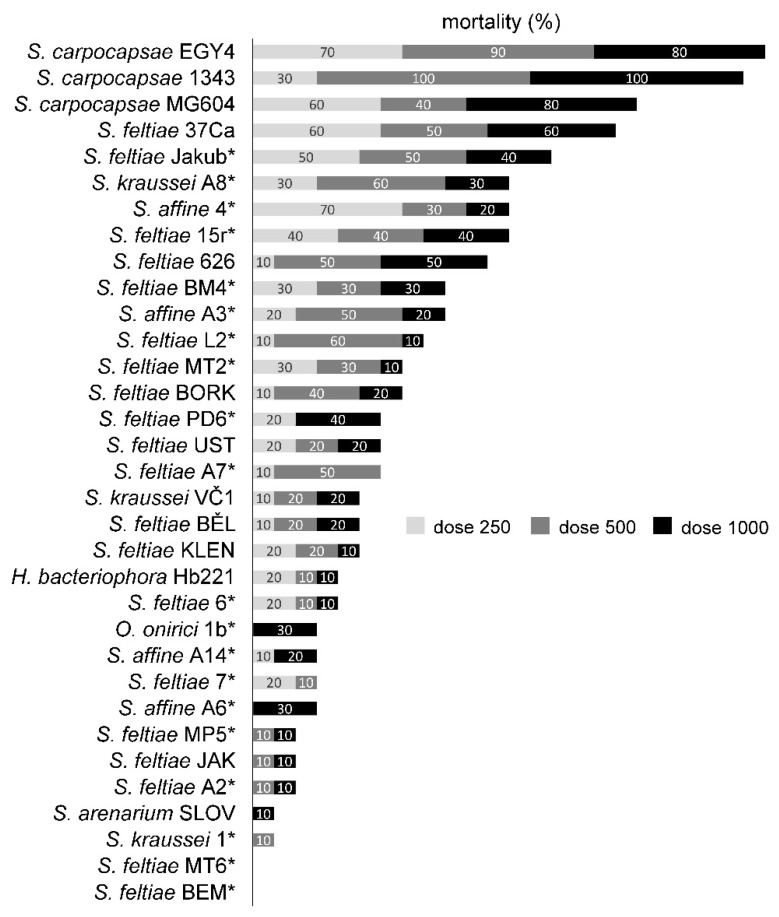
Percentage mortality of adult Colorado potato beetles caused by various EPN species and strains applied at three doses of 250, 500, and 1000 IJs per host. Endemic strains isolated within this study are marked (*).

In the experimental infections, both the nematode invasion and pathogenicity were quite variable (Figure 2 and Figure 3), with strains with negligible invasion and negligible mortality (*S. affine* A14, *S. kraussei* 1) to strongly invasive and pathogenic strains (e.g., *S. carpocapsae* MG604). However, some strains with moderate invasion caused only a very low mortality (e.g., *S. feltiae* Jakutsk). Overall, there was no difference in invasion between endemic and non-endemic nematodes (F = 0.404, *p* = 0.527, df = 1) but non-endemic nematodes caused significantly higher mortality of CPB (F = 6.396, *p* = 0.013, df = 1).

In general, *S. carpocapsae* strains had consistently moderate to high invasion and caused the highest CPB mortality reaching 100% in higher doses of *S. carpocapsae* strain 1343. Performance of *S. feltiae* and *S. kraussei* strains was variable, and the highest mortality, reaching 50% in all doses, was observed in *S. feltiae* 37Ca and *S. feltiae* Jakub, with the latter showing a higher invasion. The invasiveness and pathogenicity of *S. affine* strains and the only heterorhabditid strain, *Heterorhabditis bacteriophora* Hb221, were lower. The facultatively parasitic nematode *Oscheius onirici* caused CPB mortality only at the highest dose, and its invasion was very high, but occurred only in the dead beetles.

Based on these results, two EPN strains, *S. carpocapsae* 1343 and *S. feltiae* Jakub, were selected for further experiments.

### 3.3. Pot Experiments

**Figure 4:**
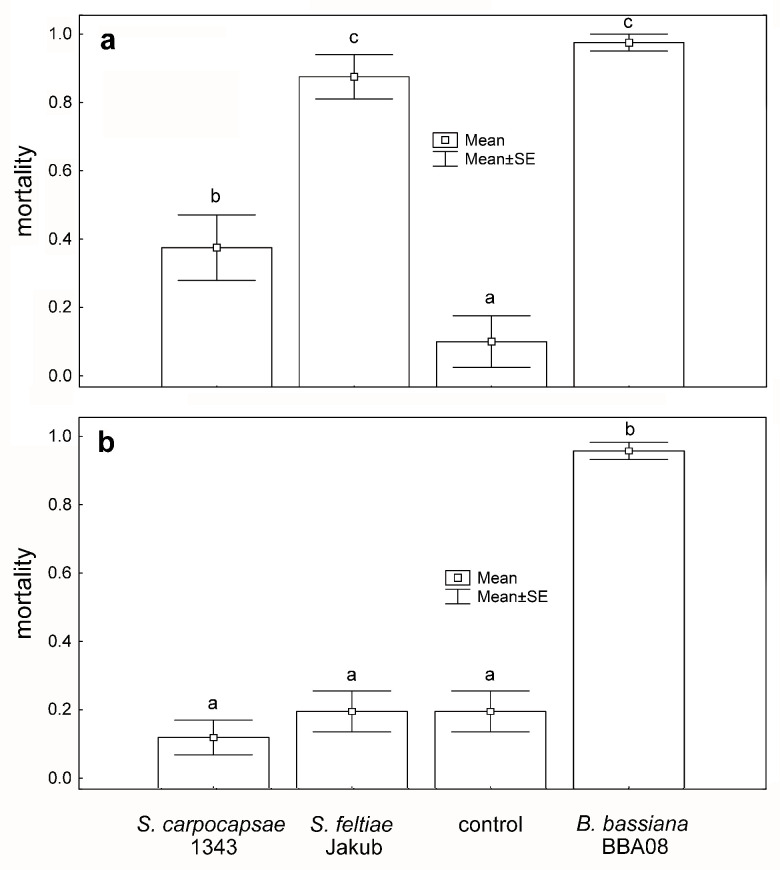
Mortality of pupating CPB in the soil (**a**) and adults on the potato leaves (**b**) after the application of entomopathogenic nematodes and fungus. Different letters above bars indicate statistically significant differences.

Soil application of both nematodes (*S. carpocapsae* 1343 and *S. feltiae* Jakub) and fungus resulted in a significant decrease in the number of emerging CPB adults in comparison to control (df = 3, 28, F = 31.9, *p* ˂ 0.001). As visible in the graph (Figure 4a), both nematode strains and fungus caused significantly higher mortality than was observed in control (*p* ˂ 0.05). *Beauveria bassiana* and *S. feltiae* Jakub have significantly (*p* ˂ 0.05) higher mortality (more than 80%) than *S. carpocapsae* 1343 (less than 40%).

After the application of nematodes on the leaves against adults of *L. decemlineata,* the observed mortality was not significantly different from control and reached a maximum of around 20% or lower (Figure 4b). On the other hand, the leaf application of fungus (df = 3, 28, F = 37.5, *p* ˂ 0.001) was very successful, reaching mortality higher than 90%.

### 3.4. Field Application

In the field experiment, statistical analyses revealed significant differences among treatments (F = 3.15, *p* = 0.025, df = 3), but only the application of fungus *B. bassiana* significantly decreased the number of emerging CPB adults in comparison to control sites, by ca. 30% (*p* = 0.014) with a mean number of CPB adults emerged per 1 potato plant of 25.9 ± 2.84 vs. 35.7 ± 3.43 in control plots. The number of emerging adults from the sites with the application of *S. feltiae* strain Jakub (29.6 ± 3.24) and its combination with fungus *B. bassiana* (29.6 ± 2.12) were around 20% lower in comparison to control (35.7 ± 3.43), though the difference was not statistically significant (*p* = 0.014).

## 4. Discussion

Endemic EPN strains can be more effective against target pests than exotic ones [29,30], and, therefore, we performed the sampling in the target localities. Interestingly, we recorded a massive presence and diversity of EPNs in Czech potato fields. Entomopathogenic nematodes are ubiquitous soil organisms; however, in general, their occurrence in the agroecosystems tends to be low [31]. In the light of this fact, our results are surprising, and it can be assumed that, in Czech fields, naturally occurring EPNs could contribute to the regulation of CPB populations.

The ubiquity of *S. feltiae* and to a lesser extent *S. affine* is not surprising, as the former species is the most common EPN in the Czech Republic and the latter is frequent in the agroecosystems [32]; on the other hand, the isolation of two strains of *S. kraussei* is surprising, as it represents the first finding of this species in arable areas of the Czech Republic [32]. The isolation of *Oscheius onirici* is the first finding of this nematode in the Czech Republic.

Our experimental infections with endemic and non-endemic strains did not support the superiority of endemic strains, but conversely, non-endemic EPN strains were generally more pathogenic. In accordance with our results, Berry et al. [33] observed exotic EPN strains being superior to endemic ones in CPB infections.

Overall, the experimental infections have shown considerable variability both in the invasion and pathogenicity of the nematodes tested. Inter and intraspecific differences in EPN infectivity towards different hosts are well known (e.g., [34]), and in this particular case, the differences could be related to host immune response. The Colorado potato beetle is known to possess an effective immunity response towards EPNs via encapsulation [35]. Accordingly, during dissections, we often observed dead IJs or even adults encapsulated by CPB hemocytes. Different insects have been shown to differ in their immune reaction towards different EPN species [36]; it can be hypothesized that the high effectivity of *S. carpocapsae* strains could be due to the lower immune response of the host. The high variability in the performance of nematode species and strains highlights the importance of thorough screening in the search for suitable biocontrol agents for CPB control.

With a majority of the tested strains killing only ca. 30% of CPB adults or less, even at the highest dose of 1000 IJs per beetle, the mortality is quite low in comparison with published data from infections of CPB prepupal stages [20,33], but CPB adults are known to be less susceptible than prepupal stages [16]. Trdan et al. [15] reported the lowest LC50 for adult CPB infections being 463 IJs/adult in *S. carpocapsae*, while in the present study, a mortality of well over 50% was achieved with *S. carpocapsae* strains even at the lowest dose of 250 IJs per one CPB adult.

Field applications of biocontrol agents tend to be less successful due to uncontrolled abiotic and biotic factors. The results of the field application of the nematodes, fungus, and their combination with only the fungus causing a significant decrease in comparison to untreated control are not surprising. The rate of infestation in the experimental field was very high, with more than 200 adult beetles emerging from under a canopy of a single plant, so possibly the standard doses of bioagents were too low to cause considerable effect. In comparison to similar studies, the 20% decrease of the CPB population in *S. feltiae*–treated sites in our study was lower than the 31% reduction of the late-season adults of the CPB treated with *S. carpocapsae* at a little higher dosage (7.6 × 10^5^ per m^2^ vs. 5 × 10^5^ per m^2^) [37]. Caged small plots with potatoes treated with *S. feltiae* and *H. heliothidis* (later synonymized with *H. bacteriophora*) decreased the emergence of CPB adults by 66–77% [38], but the dose of 90–150 IJs per cm^2^ was two to three times higher than in the present study.

Interestingly, in our experiment, the combination of both bioagents had a worse result than fungus alone. Interactions between some bioagents can be antagonistic [39], and, similar to our result, Shapiro-Ilan et al. [40] observed that, when pairs of nematode and fungal pathogens attacked weevils of *Curculio caryae*, most pairings were less effective than a single highly effective entomopathogenic species. The negative effect of entomopathogenic fungi on EPNs has been described on several occasions. The development of *Steinernema feltiae* was negatively affected when the nematode was applied on Colorado potato beetle larvae 24 h or later after fungus *I. fumosorosea* [20]. A similar negative effect on nematode growth was observed in the interactions between the EPF *Metarhizium anisopliae* and EPNs *Steinernema glaseri* [22] and *H. bacteriophora* [41]. On the other hand, in our study, the combined application showed worse results in comparison to fungus alone, which would suggest a negative effect of the nematode on fungus performance. The secondary metabolites of the nematode bacterial symbionts of the genus *Xenorhabdus* produce many bioactive compounds, including fungistatic substances [42], and a negative effect of some of these metabolites on the growth of the fungus *B. bassiana* were observed [23]. Coinfections of CPB adults in the locality thus could reduce the reproduction of the fungus, leading to lower overall CPB mortality. Further research is needed to shed more light on the interactions of these particular biocontrol agents. Nevertheless, our present results do not allow us to recommend the combination of the nematodes and *B. bassiana* in the field.

Pupating summer generation CPB adults spend only several weeks in the soil, and thus the time that biocontrol agents have to localize and infect pupating insects is limited. Our further research will focus on overwintering CPB generation, where the nematodes and fungi can operate for a long period.

## 5. Conclusions

In conclusion, we demonstrate that, due to the high variability in nematode pathogenicity towards CPB adults, large-scale screening is necessary in order to select the effective nematode strains. The selected nematodes and fungus *B. bassiana* effectively decreased the emergence of second-generation CPB adults in pots, while the effect of the field application of both agents and their combination was low. Further research will focus on overwintering CPB generation.

## Figures and Tables

**Table 1:** Entomopathogenic nematodes used in different experiments. Marked strains (*) were isolated within this study, other strains originate in the laboratory collection.

Species	Strain	Locality	Habitat
*S. kraussei, Oscheius onirici*	1 *, 1b *	Dvorce, Lysá nad Labem	field edge
*S. affine*	4 *	Zubří, Nové Město n. M.	field
*S. feltiae*	6 *	Zubří, Nové Město n. M.	field
*S. feltiae*	7 *	Oupor, Obříství	field edge
*S. affine*	8 *	Havl. Borová	field
*S. feltiae*	15 *	Oupor, Obříství	field edge
*S. feltiae*	A2 *	Malý Bor, Klatovy	field
*S. affine*	A3 *	Dřevíkov, Chrudim	field edge
*S. affine*	A6 *	Malý Bor, Klatovy	field edge
*S. feltiae*	A7 *	Supíkovice	field edge
*S. kraussei*	A8 *	Dřevíkov, Chrudim	field edge
*Rhabditis terricola*	A9 *	Chlebovice, Frýdek-Místek	field
*Diplogaster* sp.	A11 *	Březová, Opava	field edge
*Diplogaster* sp.	A12 *	Supíkovice	field edge
*S. affine*	A13 *	Supíkovice	field
*S. affine*	A14 *	Valečov	field edge
*S. feltiae*	Jakub *	Zbudov, Dívčice	field edge
*S. feltiae*	BM4 *	Bojanovice	field edge
*S. feltiae*	BEM *	Bělčice	field edge
*S. feltiae*	BP6 *	Bělčice	field
*S. feltiae*	L2 *	Horažďovice	field edge
*S. feltiae*	MP5 *	Malonty	field
*S. feltiae*	MT2 *	Malonty	field
*S. feltiae*	MT6 *	Malonty	field
*H. bacteriophora*	HB221	Czech Republic	-
*S. arenarium*	SLOV	Slovakia	-
*S. carpocapsae*	1343	Czech Republic	-
*S. carpocapsae*	Egy4	Egypt	-
*S. carpocapsae*	MG604	Switzerland	-
*S. feltiae*	626	Czech Republic	-
*S. feltiae*	37Ca	Canada	-
*S. feltiae*	Bork	Czech Republic	-
*S. feltiae*	Jakutsk	Russia	-
*S. feltiae*	klen	Czech Republic	-
*S. feltiae*	NFUST	Russia	-
*S. feltiae*	Běl	Belarus	-
*S. kraussei*	VČ1	Czech Republic	-

## Data Availability

Not applicable.
